# Analysis on the Path of Digital Villages Affecting Rural Residents' Consumption Upgrade: Based on the Investigation and Research of 164 Administrative Villages in the Pilot Area of Digital Villages in Zhejiang Province

**DOI:** 10.1155/2022/9928030

**Published:** 2022-08-29

**Authors:** Liying Zhang, Xingchao Ma

**Affiliations:** Zhejiang Normal University, Jinhua, Zhejiang, China

## Abstract

The weak consumption of rural residents has become an important factor restricting the healthy development of the Chinese economy. With the continuous development of digital construction in rural areas, its role in the upgrading of rural residents' consumption is becoming more and more important. Based on the mechanism of 164 administrative villages in the pilot area of Zhejiang Province, and using staggered cluster analysis, MLR analysis, mediation effect model, etc., this paper discusses the impact and transmission mechanism of digital villages on rural residents' consumption upgrade. The results show that farmers' wage income, property income, and total income all play a significant positive mediating role in the process of promoting consumption upgrading in digital villages; information literacy is a latent variable in the process of promoting consumption upgrading in digital villages. It also played a significant positive mediating role. The research provides the micromechanism of the digital village affecting the consumption upgrade of rural residents and puts forward to promote the process of infrastructure construction, improve the construction of e-commerce system, pay attention to the differences among residents and other suggestions.

## 1. Introduction

As one of the three troikas driving the national economy, consumption is the main driving force for the transformation of China's economic structure and the realization of endogenous economic growth. With the successive proposals of China's rural revitalization strategy and the dual-cycle strategy with the domestic market as the mainstay, it is of great significance to effectively activate the blue ocean of rural consumption and fully release the consumption potential of rural residents to empower rural development and smooth the domestic economic cycle. However, according to statistics, the total consumption of rural residents, who account for nearly 40% of China's total population, only accounts for about 20% of the total consumption, and the consumption potential of rural residents has not been fully released.

Since the 18th National Congress of the Communist Party of China, the central government has attached great importance to digital construction, especially the digital construction of agriculture and rural areas, and it has also made strategic arrangements for digital construction many times. Accelerating the construction of rural infrastructure focusing on new infrastructure construction provides digital empowerment for expanding rural consumption and also provides a new perspective and opportunity for this paper.

The marginal contribution of this paper is that the existing research mainly focuses on the development and transformation of rural industries under the digital village policy, but few scholars have explored the impact of digital villages on the consumption upgrade of rural residents. From the perspective of digital villages, this paper uses the degree of policy understanding, policy participation, and policy influence as measurement indicators to quantify digital village policies and innovatively explore the relationship between digital villages and rural residents' consumption upgrades.

## 2. Literature Review and Research Hypotheses

With the proposal of the digital village policy, the impact of digital technology on the consumption of rural residents has become a new focus of academic attention. While promoting the industrial productivity and sustained economic growth [1]; digital technology profoundly affects people's living habits and behaviors [2]; which in turn affects and changes the consumption concept, consumption pattern, and consumption structure of rural residents [3]. So, this paper draws the following hypothesis:

  H1: the digital village has a significant positive impact on the consumption upgrade of rural residents.

Rural households often reduce risk by diversifying their income. The impact of the rural digital economy on income is a mixture of agricultural income such as agricultural product prices, output, sales, and nonagricultural income such as employment opportunities and wages [4]. Digital technology promotes rural economic development and farmers' income levels by enhancing the mobility of factors between urban and rural areas [5]. Farmers' income is specifically divided into four parts: farmers' operating income, wage income, property income, and transfer income [6]. The digital village policy is conducive to encouraging rural labor to start businesses and employment, provides employment guidance and guarantees, promotes the continuous transfer of rural labor to nonagricultural industries, and vigorously promotes the continuous growth of farmers' wage income, property income, and transfer income [7].

Regarding the impact of income on consumption, Flavin [8] believes that people's consumption has a strong correlation with their future expected income and concludes that consumption is “excessively sensitive” to income. Zeldes [9] affirmed the influence ability of stochastic fluctuation income on consumption decisions by studying the influence of stochastic fluctuation income on consumption optimization behavior. From this research results, this paper draws the following hypothesis:

  H2: the total income of rural residents, wage income, transfer income, property income, and household business income are the transmission paths of digital rural areas to promote the upgrading of rural residents' consumption.

Initially, only a few foreign scholars conducted a simple exploration of farmers' information literacy. Mohamedali [10] pointed out that agricultural development needs to educate agricultural practitioners to obtain information. Ejedarifu [11] pointed out that the cheapest input for rural development is knowledge. With the continuous and in-depth development of different policy environments, domestic and foreign scholars and related research institutions have begun to pay attention to the cultivation of farmers' information literacy. Taking the information process as a framework, combining rural environmental changes and farmers' own development requirements, they analyze information needs from the perspective of information literacy theory, information acquisition, information transmission, information cognition, information regeneration, and information effect, and then take these as the content elements of information literacy that farmers need to cultivate [12]. As for the influence of digital countryside on information literacy, some scholars have pointed out that digital countryside can improve rural residents' participation in the digital economy through the penetration of the Internet [13]. Farmers can obtain various important information about agriculture through the Internet, learn about relevant agricultural policies in time, and reduce the degree of information asymmetry. Regarding the impact of information literacy on consumption upgrades, Nakasone et al.'s [14] research found that financial information literacy significantly improved household consumption expenditure and consumption propensity. Meng and Yan [15] believe that household information literacy significantly increases the total rural household consumption expenditure and service consumption expenditure. Therefore, this paper proposes the following hypothesis:

  H3: the improvement of rural residents' information literacy is the transmission path for the digital village to promote rural residents' consumption upgrade.

## 3. Data Source, Variable Selection, and Model Construction

### 3.1. Data Sources

The scope of this study covers 164 villages in Lin'an District, Cixi City, Pinghu City, and Deqing County. The formal survey adopts the form of random distribution of questionnaires, and the questionnaires are divided into two parts: objective questions and subjective questions. The objective question is to investigate the basic information of local residents, including occupation and education level. The subjective question focuses on the construction of digital villages, the influence mechanism of rural residents' consumption, and the consumption of rural residents. The questionnaires were divided into self-administered questionnaires and substitute-filled questionnaires. Elderly rural residents were assisted by members of the group to fill in, while the rest of the subjects completed the questionnaires by themselves. In view of the research requirements to ensure the effective recovery rate of the questionnaires, a total of 1204 questionnaires were issued, and the final questionnaire was recovered and confirmed to be 1034 valid copies, with an effective questionnaire rate of 85.88%. The valid questionnaire will also be used as the original data of the later writing report.

### 3.2. Variable Selection

#### 3.2.1. Control Variable-Basic Information of Villagers

The control variables in this paper include whether they have purchased social insurance, whether they have access to broadband networks, and villagers' consumption concepts. Among them, whether they have purchased social insurance (Insurance Condition) is used to control the impact of whether you have paid social insurance fees on the consumption upgrade. If they pay social insurance fees, it will inevitably lead to a decrease in income, which may affect consumption upgrades; whether to access broadband networks (Broadband) is used to control the impact of Internet use on villagers' consumption upgrade; the consumption concept is used to control the impact of different consumption concepts on villagers' consumption upgrade.

#### 3.2.2. Independent-Digital Rural Dimension

Digital village is the application of networking, informatization, and digitization in agricultural and rural economic and social development. It is also a process of modernization and transformation of agricultural and rural areas that is endogenous to the improvement of farmers' modern information skills. In view of this, this paper refers to the summary of the digital village by Zeng et al. [16]; and investigates the digital village from the following three dimensions: (1) Policy understanding: whether residents pay attention to the local digital village policy; (2) Policy participation: whether villagers participate in the construction of digital villages and whether to promote digital village policies; (3) Policy influence: whether villagers are affected by digital village policies.

#### 3.2.3. Dependent Variable-Consumption Upgrade Dimension

Consumption upgrade is usually manifested in the improvement of consumption level, the improvement of consumption quality, the strengthening of consumer interest protection, and the structural upgrade and level improvement of various consumption expenditures in the total consumption expenditure. Referring to the processing method of Kong and Li [17]; this paper investigates the consumption upgrade from the two dimensions of rural residents' consumption habits (Habit) and rural residents' consumption structure (Structure). Consumption structure refers to the proportion of various consumption expenditures in total expenditures. The consumption structure of rural residents is embodied in the proportion of rural residents' expenditure in basic consumption and high-end consumption [18]. With the continuous increase of income and the change of consumption concept, rural residents tend to pursue higher-quality consumption, and the level of their consumption structure has gradually improved. Consumption habit refers to a behavioral manifestation of the consumer's stable consumption preference for a certain consumer object formed in the process of long-term consumption practice by the main consumer. The consumption habits of rural residents are embodied in the characteristics of villagers buying products, receiving services, and changes in consumption patterns [19].

#### 3.2.4. Intermediation Variable-Information Literacy Dimension

This paper mainly refers to the classification of information literacy by Yan et al. [20] and measures the information literacy level of rural residents from the following two indicators: (1) Information ability (Skill), which is divided into information acquisition ability, information understanding ability, and Information sharing ability which is embodied in the use of computer networks, office software and mobile payment by rural residents; (2) Information awareness (Recognization), which is specifically divided into two aspects: information value awareness and information demand awareness.

#### 3.2.5. Mediation Variable-Income Level Dimension

Villagers' consumption expenditure is closely related to their disposable income. The current income increase will promote the basic consumption and restrain the consumption of high-end goods; persistent income increase will restrain the consumption of basic goods and promote the consumption of high-grade goods [21]. Referring to the handling method of Wei and Venayagamoorthy [21]; this paper examines the income level from the following two dimensions: (1) wage income (Wage), which is reflected in the impact of the increase in villager wage income on consumption upgrading; (2) nonwage income, including operating income (operating), property income (property), and transfer income (transfer), is embodied in the impact of the growth of villagers' nonwage income on consumption upgrades.

### 3.3. Model Building

#### 3.3.1. The Multiple Linear Regression Model of the Digital Village to Promote Rural Residents' Consumption Upgrade

Based on the consideration of literature and field research, this paper decides to first use the multiple linear regression model to explore the effect of the digital village and its subdimensions on the consumption upgrade of rural residents. The specific regression equation constructed in this paper is as follows:

(1) $$\begin{array}{c} \left\{\begin{array}{c} C\_\text{Structure}=\alpha_{1}D\_\text{Village}+\sum_{\sigma -2}^{n}\text{Control}\cdot \alpha_{\sigma }+\beta ,\\ C\_\text{Habit}=\gamma_{1}D\_\text{Village}+\sum_{\sigma =2}^{n}\text{Control}\cdot \gamma_{\sigma }+\delta , \end{array}\right. \end{array}$$

where C_Structure is the consumption structure, C_Habit is the consumption habit, D_Village is the digital countryside, and Control is the control variable.

#### 3.3.2. The Mediating Effect Model of Digital Villages to Promoting Rural Residents' Consumption Upgrade

This paper takes the rural residents' income as the intermediary variable and divides the income into five categories: wage income level, transfer income level, property income level, operating income level, and total income level. This paper uses the Bootstrap method [1] to test the intermediary role of the income level in the impact of digital rural areas on the consumption upgrading of rural residents.

Meanwhile, in order to ensure the robustness of the test results of parallel mediation effects, process is used to construct a custom path model.

#### 3.3.3. Structural Equation Model of Digital Village to Promote Rural Residents' Consumption Upgrade

In this paper, confirmatory factor analysis was carried out on 4 latent variables and 15 observed variables. The structural validity test is shown in Table 1. All model fitting indicators meet the standards.

It can be seen from Table 2 that the factor loads of all latent variables in the model are all greater than 0.7, indicating that each latent variable has a good representativeness for the topic to which it belongs. In addition, the average variance variation AVE of each latent variable is greater than 0.5, and the combined reliability CR is greater than 0.75, indicating that the convergent validity is ideal.

## 4. Results and Analysis

### 4.1. Descriptive Statistical Analysis

Based on 1034 valid samples, this paper conducts a descriptive analysis from the perspective of demographic characteristics. In terms of gender, in order to avoid the specific situation of the respondents in the questionnaire, during the investigation process, we avoided crowds in gathering places and tried our best to choose people who walked freely and storefronts along the street for the investigation. The final descriptive statistics of basic information are shown in Table 3.

### 4.2. Regression Outcome Analysis

This paper uses SPSS23.0 to establish a multiple regression model and introduces independent variables, dependent variables, and control variables for analysis. The results obtained are shown in Table 4:

Therefore, the table above can obtain

(2) $$\begin{array}{c} C\_\text{Structure}=0.696\cdot D\_\text{Village}+\sum_{\sigma =2}^{n}\text{Control}\cdot \alpha_{\sigma }+1.295,\\ C\_\text{Habit}=0.696\cdot D\_\text{Village}+\sum_{\sigma =2}^{n}\text{Control}\cdot \gamma_{\sigma }+1.635. \end{array}$$

That is, after controlling for the interference of all control variables such as gender, age, occupation, education level, health status, insurance status, annual income, family size, household broadband access, and location, digital villages can significantly promote rural residents' consumption structural upgrade (=0.696, *p* < 0.001) and it can also significantly improve the consumption habits of rural residents (=0.654, *p* < 0.001).

### 4.3. Heterogeneity Analysis

#### 4.3.1. Grouped by Region

The impact of the digital village on rural residents' consumption upgrading may be different in different pilot areas, which may be reflected in the difference in the actual implementation of this policy in different regions. Based on this, this paper divides all data into four categories according to different pilot areas. The results of the regression models in different regions are shown in Tables 5 and 6:

As can be seen from the table, digital villages have a significant effect on promoting the consumption upgrading of rural residents in different pilot areas. Specifically, on the one hand, the digital countryside promoted the upgrading of rural residents the most (*p* < 0.01), Pinghu County second (*p* < 0.01), Cixi City third (*p* < 0.01), Deqing County smallest (*p* < 0.01); on the other hand, the digital countryside on the Pinghu County pilot rural residents consumption habits change (*p* < 0.01), Cixi City second (*p* < 0.01), Deqing County third (*p* < 0.01), and Linan District the smallest (*p* < 0.01). *α*_1_=0.75*α*_1_=0.72*α*_1_=0.68*α*_1_=0.59*β*_1_=0.71*α*_1_=0.68*α*_1_=0.59*α*_1_=0.37

#### 4.3.2. Grouped by Age

There are great differences in the acceptance of digital technology by different age groups. This paper divides the surveyed rural residents into young people (under 40 years old, excluding 40 years old) and middle-aged and elderly people (over 40 years old). The regression results are shown in Tables 7 and 8:

As can be seen from the above table, the digital countryside can significantly promote the upgrading of the consumption structure of rural residents of all ages and also have a significant positive impact on the change of rural residents' consumption habits. Among them, the positive effect of the digital countryside on the consumption upgrading of groups under 40 is greater than that on groups aged 40 and over.

#### 4.3.3. Grouped by Gender

There are great differences in the consumption concepts among the different genders, which may also lead to the differences in the consumption structure and consumption habits of the different gender groups in the digital villages. Based on this, all the subjects were divided into men and women for separate regression analysis, and the results are shown in Tables 9 and 10:

As can be seen from the above table, digital villages can have a significant impact on the consumption structure and consumption habits of different gender groups, and the promotion of the consumption structure of male groups is greater than that of women.

### 4.4. Mediating Effect Test Results

It can be seen from Table 11 that the confidence interval of Bootstrap test for wage income level, property income level, and total income level does not contain 0. At a 95% confidence level, the confidence interval of Bootstrap test for both transfer income level and operational income level is 0. Therefore, the wage income level, property income level, and total income level of rural residents are the transmission path of digital rural policy to promote the upgrading of rural residents' consumption structure.

As shown in Table 12, the mediating effect of total income level is significant. Therefore, the wage income level, property income level, and total income level of rural residents are the transmission path of digital rural policies to promote the improvement of rural residents' consumption habits.

### 4.5. Empirical Analysis Results

**Figure 1:**
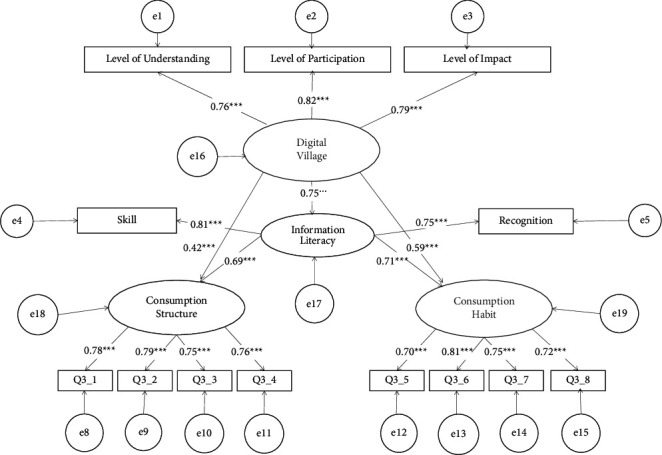
Schematic diagram of structural equation model results.

Figure 1 shows the standardized path coefficient and mediating effect test of “Digital countryside-Information literacy-Consumption upgrade” in this paper. As can be seen from the figure, the standardized path coefficient of information literacy in the digital countryside is 0.75, the standardized path coefficient of information literacy on consumption structure is 0.69, while the standardized path coefficient of digital villages is 0.42, then the path coefficient of information literacy that indirectly affects the consumption structure is 0.52. The standardized path coefficient of consumption habits in digital villages is 0.59, and the standardized path coefficient of information literacy on consumption habits is 0.71, so the path coefficient of digital rural information literacy that indirectly affects consumption habits is 0.53.

## 5. Main Conclusions and Policy Recommendations

Through descriptive statistics, it is preliminarily found that there is a positive transmission relationship between digital countryside and consumption upgrading. Four types of character portraits are depicted using hierarchical clustering analysis, forming a more comprehensive understanding of the sample characteristics; in terms of the quantitative analysis, in this paper, the integrated use of multiple linear regression, the intermediary effect model, and structural equation model type solid conclusions: First, information literacy is the intermediary of the digital village to promote the rural residents' consumption upgrade path, still exist in the current rural information access to a single, villagers low demand, such as the weak part of the villagers' autonomous learning ability. Second, the digital countryside can promote the consumption upgrading of rural residents through the intermediary path of “rural residents' income.” As the majority of farmers' income, operational income becomes the main factor influencing the consumption upgrading effect of rural residents in the digital countryside. Third, the mediating effect of “transfer income” in rural residents' income is not significant. Most rural residents do not have transfer income and have no influence on consumption. Some rural residents with transfer income are not clear about subsidy standards and regulations. Fourthly, digital countryside plays a more significant role in the consumption upgrading of young people in rural areas, but not in the transformation of consumption habits of middle-aged and old people.

Based on this, this paper puts forward the following policy recommendations: first, promote the process of rural infrastructure construction and improve the information literacy of rural residents by comprehensively covering the project of bringing information into villages and households, stimulating farmers' demand for information learning and strengthening rural residents' independent learning ability; second, by encouraging farmers to start their own businesses, training rural e-commerce innovation talents and focusing on the mining of rural local characteristic resources to improve the construction of rural e-commerce system, and further stimulate farmers' operating income potential; third, by unifying the standard of comprehensive income subsidies, improving the regulations on special agricultural subsidies, and effectively increasing financial support for agriculture, we should improve the system of transfer payments to support agriculture and boost the consumer confidence of rural residents. Fourthly, by improving rural residents' participation in government affairs, implementing information popularization and propaganda, and increasing the training and guidance for the elderly and other key groups, the differences in access to information can be narrowed.

## Figures and Tables

**Table 1:** Structural validity test table.

Metric	*X * ^2^/d*f*	GFI	RMSEA	CFI	NFI	NNFI
Value	2.14	0.91	0.08	0.93	0.94	0.95
Standard	<3	>0.9	<0.10	>0.9	>0.9	>0.9
Fitting results	Ideal	Ideal	Ideal	Ideal	Ideal	Ideal

**Table 2:** Results of observed variables.

Variables	*M* (SD)	*β*	AVE	CR
Digital village				
Understanding	2.86 (0.65)	0.73^*∗∗∗*^	0.63	0.84
Participation	2.75 (0.74)	0.84^*∗∗∗*^		
Influence	3.55 (0.66)	0.81^*∗∗∗*^		

Information literacy				
Skill	4.00 (0.93)	0.84^*∗∗∗*^	0.55	0.83
Recognization	4.07 (0.72)	0.77^*∗∗∗*^		

Consumption structure				
Q3_1	3.50 (0.77)	0.78^*∗∗∗*^	0.61	0.86
Q3_2	3.32 (0.84)	0.82^*∗∗∗*^		
Q3_3	3.53 (0.79)	0.75^*∗∗∗*^		
Q3_4	3.35 (0.75)	0.76^*∗∗∗*^		

Consumption habit				
Q3_5	3.55 (0.85)	0.68^*∗∗∗*^	0.56	0.84
Q3_6	3.42 (0.87)	0.84^*∗∗∗*^		
Q3_7	3.57 (0.89)	0.75^*∗∗∗*^		
Q3_8	4.00 (0.93)	0.72^*∗∗∗*^		

**Table 3:** Descriptive statistics of basic information.

Elementary item	Content	Frequency	Percentage (%)
Gender	Man	576	55.71
	Woman	458	44.29

Age	<40 years old	665	64.31
	≥40 years old	369	35.69

Education background	Completely uneducated	5	0.48
	Primary school	69	6.67
	Junior middle school	278	26.89
	High school (vocational high school, high technology)	343	33.17
	Special school	45	4.36
	Junior college	178	17.21
	Bachelor degree or above	116	11.22

Insurance status	Have bought	810	78.34
	Did not buy	224	21.66

Family size	1	8	0.78
	1–3	215	20.79
	3–5	699	67.60
	More than 5	112	10.83

Broadband	Yes	978	94.58
	No	56	5.42

Annual income	Below 6w	88	8.51
	6w–9.6w	304	29.40
	9.6w–13.2w	359	34.72
	13.2w–16.8w	176	17.02
	Above 16.8w	107	10.35

Area	Lin'an district, Hangzhou	398	38.49
	Cixi city, Ningbo city	324	31.34
	Pinghu city, Jiaxing city	157	15.18
	Deqing county, Huzhou city	155	14.99

**Table 4:** Results of the multiple regression analysis.

Dependent variable: **C**_**Structure**			Dependent variable: **C**_**Habit**		
Constant	*β*	1.295	Constant	*δ*	1.635
		(0.183)			(0.217)

Independent variable	D_Village	0.696^*∗∗∗*^	Independent variable	D_Village	0.654^*∗∗∗*^
		(0.057)			(0.068)
Controlled variable	YES		Controlled variable	YES	
*R* ^2^		0.52	*R* ^2^		0.41
*F*		148.47^*∗∗∗*^	*F*		93.38^*∗∗∗*^

Note: due to space limitation, all control variables are not listed in the regression results, the same below.

**Table 5:** Regression analysis of consumption structure upgrading of rural residents in different regions.

Lin'an district			Cixi city		
Independent variable	*D*_Village	0.75^*∗∗∗*^	Independent variable	*D*_Village	0.68^*∗∗∗*^
		(0.11)			(0.04)
Controlled variable	YES		Controlled variable	YES	
*R* ^2^		0.45	*R* ^2^		0.52
*F*		45.53^*∗∗∗*^	*F*		3.12^*∗∗∗*^

Pinghu city			Deqing county		
Independent variable	*D*_Village	0.72^*∗∗∗*^	Independent variable	*D*_Village	0.59^*∗∗∗*^
		(0.07)			(0.11)
Controlled variable	YES		Controlled variable	YES	
*R* ^2^		0.60	*R* ^2^		0.73
*F*		104.12^*∗∗∗*^	*F*		13.34^*∗∗∗*^

**Table 6:** Regression analysis table of the improved consumption habits of rural residents in different regions.

Lin'an district			Cixi city		
Independent variable	*D*_Village	0.37^*∗∗∗*^	Independent variable	*D*_Village	0.68^*∗∗∗*^
		(0.11)			(0.04)
Controlled variable	YES		Controlled variable	YES	
*R* ^2^		0.18	*R* ^2^		0.62
*F*		5.08^*∗∗∗*^	*F*		24.80^*∗∗∗*^

Pinghu city			Deqing county		
Independent variable	*D*_Village	0.71^*∗∗∗*^	Independent variable	*D*_Village	0.59^*∗∗∗*^
		(0.06)			(0.11)
Controlled variable	YES		Controlled variable	YES	
*R* ^2^		0.57	*R* ^2^		0.60
*F*		91.60^*∗∗∗*^	*F*		5.98^*∗∗∗*^

**Table 7:** Regression analysis table of consumption structure upgrading among rural residents of different ages.

Age	＜40		≥40	
Independent variable	D_Village	0.732^*∗∗∗*^	D_Village	0.575^*∗∗∗*^
		(0.07)		(0.09)
Controlled variable	YES		YES	
*R* ^2^	0.55		0.38	
*F*	99.89^*∗∗∗*^		34.29^*∗∗∗*^	

**Table 8:** Regression analysis table of the improved consumption habits of rural residents of different ages.

Age	＜40		≥40	
Independent variable	D_Village	0.846^*∗∗∗*^	D_Village	0.172^*∗∗∗*^
		(0.09)		(0.11)
Controlled variable	YES		YES	
*R* ^2^	0.34		0.39	
*F*	40.72^*∗∗∗*^		34.96^*∗∗∗*^	

**Table 9:** Regression analysis table of consumption structure upgrading of rural residents of different genders.

Gender	Male		Female	
Independent variable	D_Village	0.778^*∗∗∗*^	D_Village	0.638^*∗∗∗*^
		(0.09)		(0.08)
Controlled variable	YES		YES	
*R* ^2^	0.59		0.47	
F	81.64^*∗∗∗*^		69.47^*∗∗∗*^	

**Table 10:** Regression analysis table of the improved consumption habits of rural residents of different genders.

Gender	Male		Female	
Independent variable	D_Village	0.739^*∗∗∗*^	D_Village	0.596^*∗∗∗*^
		(0.10)		(0.09)
Controlled variable	YES		YES	
*R* ^2^	0.51		0.35	
F	58.79^*∗∗∗*^		41.01^*∗∗∗*^	

**Table 11:** Test table of mediating effect of influencing mechanism of consumption structure of rural residents.

Way					Effect value	Boost SE	Bias-corrected 95% CI		Result
							Lower	Upper	
Digital countryside	$\begin{array}{c} 0.64^{***}\\ ⟶ \end{array}$	Wage income	$\begin{array}{c} 0.11^{***}\\ ⟶ \end{array}$	Consumption structure	0.5291	0.028	0.0408	0.1138	Notable
Digital countryside	$\begin{array}{c} 0.65^{***}\\ ⟶ \end{array}$	Transfer income	$\begin{array}{c} 0.11\\ ⟶ \end{array}$	Consumption structure	0.0567	0.027	−0.0101	0.1208	Quiet
Digital countryside	$\begin{array}{c} 0.61^{***}\\ ⟶ \end{array}$	Property income	$\begin{array}{c} 0.19^{***}\\ ⟶ \end{array}$	Consumption structure	0.4874	0.035	0.0340	0.1687	Notable
Digital countryside	$\begin{array}{c} 0.50^{***}\\ ⟶ \end{array}$	Operating income	$\begin{array}{c} 0.27\\ ⟶ \end{array}$	Consumption structure	0.0492	0.053	−0.0958	0.1040	Quiet
Digital countryside	$\begin{array}{c} 0.58^{***}\\ ⟶ \end{array}$	General income	$\begin{array}{c} 0.20^{***}\\ ⟶ \end{array}$	Consumption structure	0.4167	0.045	0.0437	0.2131	Notable

**Table 12:** Test table of mediating effect of influencing mechanism of consumption habit of rural residents.

Way					Effect value	Boost SE	Bias-corrected 95% CI		Result
							Lower	Upper	
Digital countryside	$\begin{array}{c} 0.57^{***}\\ ⟶ \end{array}$	Wage income	$\begin{array}{c} 0.16^{***}\\ ⟶ \end{array}$	Consumption custom	0.3796	0.035	0.0204	0.1541	Notable
Digital countryside	$\begin{array}{c} 0.61^{***}\\ ⟶ \end{array}$	Transfer income	$\begin{array}{c} 0.09\\ ⟶ \end{array}$	Consumption custom	0.0446	0.029	−0.0088	0.1062	Quiet
Digital countryside	$\begin{array}{c} 0.59^{***}\\ ⟶ \end{array}$	Property income	$\begin{array}{c} 0.15^{***}\\ ⟶ \end{array}$	Consumption custom	0.3688	0.035	0.0182	0.1458	Notable
Digital countryside	$\begin{array}{c} 0.45^{***}\\ ⟶ \end{array}$	Operating income	$\begin{array}{c} 0.29\\ ⟶ \end{array}$	Consumption custom	0.0208	0.065	−0.0931	0.0078	Quiet
Digital countryside	$\begin{array}{c} 0.54^{***}\\ ⟶ \end{array}$	General income	$\begin{array}{c} 0.20^{***}\\ ⟶ \end{array}$	Consumption custom	0.1180	0.049	0.0386	0.2306	Notable

## Data Availability

The dataset can be accessed upon request.

## References

[1] Wen Z. L., Ye B. J. (2014). Analysis of mediating effect: method and model development. *Advances in Psychological Science*.

[2] Liu H., Ma W. (2017). Social interaction and family capital market participation behavior in the Internet era. *International Finance Research*.

[3] Wang Y., Wang H. (2021). The influence effect of digital countryside on online shopping for rural residents. *Circulation economy of China*.

[4] Hjort J., Poulsen J. (2019). The arrival of fast Internet and employment in africa. *The American Economic Review*.

[5] Cheng Z., Zhang J. (2019). Internet popularization and urban-rural income gap: theory and Empirical Evidence. *China’s rural economy*.

[6] Chen B. K., Lu M., Zhong N. H. (2015). How urban segregation distorts Chinese migrants’ consumption?. *World Development*.

[7] Yang L., Xu Y., Li P., Lu Y. (2021). Research on the impact factors and countermeasures of farmers’ income growth in guangdong Province. *China Economic and Trade Guide (China)*.

[8] Flavin M. A. (1981). The adjustment of consumption to changing expectations about future income. *Journal of Political Economy*.

[9] Zeldes S. P. (1989). Optimal consumption with stochastic income: deviations from certainty equivalence. *Quarterly Journal of Economics*.

[10] Mohamedali O. N. (1977). Practical agriculturists, literacy and agricultural information in east africa. *Libri - International Journal of Libraries and Information Services*.

[11] Ejedarifu E. F. (2015). Public library and information literacy programme:mainstreaming rural populace for information literacy in delta state. *Developing Country Studies*.

[12] Ma H., Pu P. (2019). Information quality chain: the multi-dimensional extension and tool intervention of information quality connotation. *Information Information*.

[13] Townsend L., Sathiaseelan A., Fairhurst G., Wallace C. (2013). Enhanced broadband Access as a solution to the social and economic problems of the rural digital divide. *Local Economy*.

[14] Nakasone E., Torero M., Minten B. (2014). The power of information: the ICT revolution in agricultural development. *Annual Review of Resource Economics*.

[15] Meng H., Yan X. (2020). The impact of financial literacy on urban household consumption —— based on data from Chinese household finance survey. *Research world*.

[16] Zeng Y., Song Y., Lin X., Fu C. (2021). A brief discussion on several issue(in Chinese).s of China’s digital rural construction. *Rural economy in China*.

[17] Kong W., Li A. (2019). Study on the impact of inclusive finance development on the consumption of rural residents. *Journal of Dongbei University of Finance and Economics*.

[18] Xie G. (2018). Performance, characteristics and countermeasures of rural residents’ consumption upgrading. *Business Economy Research*.

[19] Dong Y., Li Q., Zhang P. (2019). Analysis of the influence of inclusive finance development on the consumption upgrading of rural residents in China. *Business Economy Research*.

[20] Yan B., Zhao P., Liu T. (2021). The impact of information literacy on farmers participation in e-commerce —— is based on the intermediary role of farmers’ internal perception and the regulatory effect of government promotion. *Journal of Huazhong Agricultural University (Social Science edition)*.

[21] Wei Y., Venayagamoorthy G. K. (2017). Cellular computational generalized neuron network for frequency situational intelligence in a multi-machine power system. *Neural Networks: The Official Journal of the International Neural Network Society*.

